# Discovery of novel JAK1 inhibitors through combining machine learning, structure-based pharmacophore modeling and bio-evaluation

**DOI:** 10.1186/s12967-023-04443-6

**Published:** 2023-08-28

**Authors:** Zixiao Wang, Lili Sun, Yu Xu, Peida Liang, Kaiyan Xu, Jing Huang

**Affiliations:** 1 Department of Pharmacy, Honghui Hospital, Xi’ an Jiaotong University, Xi’ an, 710054 China; 2 Department of Pharmacy, The First Affiliated Hospital of Xi’an Jiaotong University, Xi’an, 710061 China; 3 State Key Laboratory of Natural Medicines,Jiangsu Key Laboratory of Drug Discovery for Metabolic Diseases, Center of Drug Discovery,China Pharmaceutical University, Nanjing, 210009 China; 4 School of Pharmacy, Lanzhou University, Lanzhou, 730000 China

**Keywords:** Janus kinase 1, Machine learning, Pharmacophore, Molecular dynamics simulations, Virtual screening

## Abstract

**Background:**

Janus kinase 1 (JAK1) plays a critical role in most cytokine-mediated inflammatory, autoimmune responses and various cancers via the JAK/STAT signaling pathway. Inhibition of JAK1 is therefore an attractive therapeutic strategy for several diseases. Recently, high-performance machine learning techniques have been increasingly applied in virtual screening to develop new kinase inhibitors. Our study aimed to develop a novel layered virtual screening method based on machine learning (ML) and pharmacophore models to identify the potential JAK1 inhibitors.

**Methods:**

Firstly, we constructed a high-quality dataset comprising 3834 JAK1 inhibitors and 12,230 decoys, followed by establishing a series of classification models based on a combination of three molecular descriptors and six ML algorithms. To further screen potential compounds, we constructed several pharmacophore models based on Hiphop and receptor-ligand algorithms. We then used molecular docking to filter the recognized compounds. Finally, the binding stability and enzyme inhibition activity of the identified compounds were assessed by molecular dynamics (MD) simulations and in vitro enzyme activity tests.

**Results:**

The best performance ML model DNN-ECFP4 and two pharmacophore models Hiphop3 and 6TPF 08 were utilized to screen the ZINC database. A total of 13 potentially active compounds were screened and the MD results demonstrated that all of the above molecules could bind with JAK1 stably in dynamic conditions. Among the shortlisted compounds, the four purchasable compounds demonstrated significant kinase inhibition activity, with Z-10 being the most active (IC_50_ = 194.9 nM).

**Conclusion:**

The current study provides an efficient and accurate integrated model. The hit compounds were promising candidates for the further development of novel JAK1 inhibitors.

**Supplementary Information:**

The online version contains supplementary material available at 10.1186/s12967-023-04443-6.

## Introduction

The Janus kinases (JAKs) family consists of four tyrosine kinases [JAK1, JAK2, JAK3, and tyrosine kinase 2 (TYK2)], a class of cytoplasmic tyrosine kinases associated with cytokine receptors [1]. After stimulated by cytokines, JAKs become enzymatically active and phosphorylate themselves, and then signal transducer and activator of transcription (STAT) [2, 3], which can act directly as transcription factors or trigger downstream signaling pathways [4]. JAK1 isoform is regulated by more than ten cytokine signals from interferon (IFN γ, α) receptor, gamma common (γc) subfamily and glycoprotein 130 (gp130) receptor families [5, 6]. It can phosphorylate any STAT protein (STAT 1–6) and is ubiquitously expressed in human tissues [7]. The JAK1/STAT signaling pathway dysregulated activity is mainly associated with autoimmune illnesses, acute lymphoblastic leukemia, acute myelogenous leukemia, and solid organ malignancies [8–11]. The critical role of JAK1 in the above diseases has emerged as an appealing therapeutic target and has inspired the pursuit of JAK1 inhibitors.

**Fig. 1:**
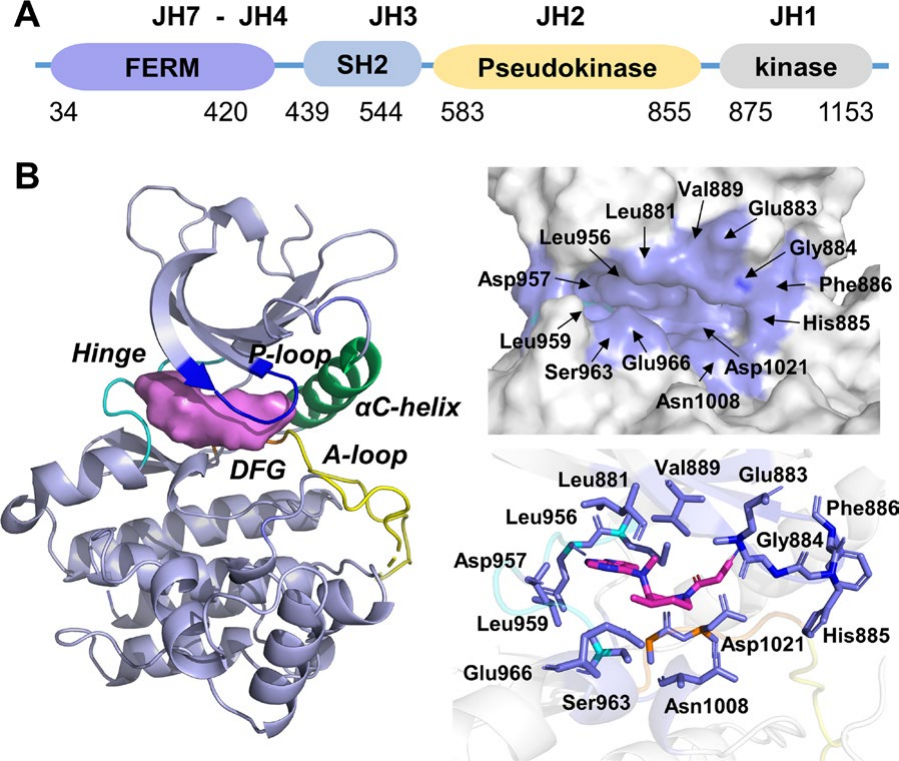
Crystal structure and active pocket of JAK1 (PDB ID: 3EYG)

Currently, approved JAK1 inhibitors including Ruxolitinib, Tofacitinib, Upadacitinib, Abrocitinib, and numerous additional second-generation inhibitors are now under investigation to treat myelofibrosis, ulcerative colitis, atopic dermatitis, and autoimmune illnesses [12–15]. However, challenges remain in developing JAK1 inhibitors, and safety and tolerability issues need to be urgently addressed [2]. Structurally, JAK1 consists of seven distinct structural domains (JH1-JH7) (Fig. 1A), with the C-terminal kinase (JH1) module having an ATP-binding site that is formed by the P-loop, A-loop, hinge region, DFG and αC-helix (Fig. 1B) [16]. All the JAK family members share a highly conserved kinase domain, particularly in the ATP-binding site, giving rise to off-target promiscuity of existing drugs and complicating the achievement of JAK isoform specificity [17, 18]. Nevertheless, conventional synthesis and screening methods are laborious, expensive, and time-consuming. Therefore, developing a robust strategy to screen novel JAK1 inhibitors with high potency is urgently needed.

Pharmacophore models describe the vital molecular features and their spatial arrangement of ligand–protein interactions and are a fast and efficient method for virtual screening (VS) active drug molecules [19, 20]. Despite the significant advances, the pharmacophore approach still faces several challenges, such as the low efficiency of screening large chemical databases with flexible molecules and high false positive/negative rates due to model quality issues [21, 22]. Machine learning (ML) [e.g., random forest (RF) and support vector machine (SVM)], especially deep neural network (DNN), as one of deep learning, have become popular after giving rise to epochal developments in the fields of computer vision and natural language processing. Its advanced algorithms and inference techniques provide fresh opportunities in various fields of data science, including biomedicine [23, 24]. Compared to traditional ML methods with manually designed features, DNN facilitates the utilization of large data sets by automatically learning features from raw input data and having fewer generalization errors [25]. Recently, sophisticated deep learning methods have been applied in VS due to their high recall and low false-positive rates, and could be combined with other methods to develop more efficient and accurate VS methods to discover novel active molecules [26–28]. However, as far as we know, research on ML predictive models for VS of kinase inhibitors was quite limited, and lacked bioactivity validation [5, 20, 29, 30]. Therefore, combining pharmacophore and ML models is necessary to build a powerful integrated model to screen potential JAK1 inhibitors.

In this study, we aimed to develop accurate integrated models to screen potential JAK1 inhibitors from an extensive compound database. To achieve this goal, we collected a highly diverse positive and negative dataset. Based on three molecular descriptors and six ML algorithms, a series of prediction ML models and pharmacophore models were constructed to screen the ZINC database. Additionally, the detailed binding modes structure–activity relationship among the hit molecules and JAK1 protein were elucidated via molecular docking and molecular dynamics (MD) simulation. Finally, some of the screened compounds were validated for in vitro biological activity.

## Materials and methods

### Data collection and preparation

The dataset of JAK1 inhibitors for VS was retrieved from the ChEMBL database (https://www.ebi.ac.uk/chembl/) (accessed Oct 2022) [31]. After removing the redundancy, 3834 inhibitors (IC_50_ ≤ 1000 nM) were collected as active set. Besides, 6590 decoys were retrieved from the DUD-E database (https://dude.docking.org/) (accessed Oct 2022) included in the inactive datasets [32]. To increase the structural diversity of inactive datasets, the Extended connectivity fingerprints 4 (ECFP4, radius = 2) of 10 million compounds from PubChem database (https://pubchem.ncbi.nlm.nih.gov/) (accessed Oct 2022) were calculated and clustered with Discovery Studio 2019 to retrieve 5640 compounds. The 5640 compounds were also included in the inactive set, resulting in total of 12,230 compounds in the inactive database. Finally, the active and inactive databases were randomly divided into test set and training set with ratio of 1:3 (Table 1).

**Table 1:** Number of compounds in datasets for ML models

	Inhibitors	Non-inhibitors	Total
Train	2875	9172	12,047
Test	959	3058	4017

### Molecular fingerprint calculation

Numerous studies have revealed that the performance of prediction models are closely related to the representation which encode the molecular features for similarity assessment in medicinal chemistry [33]. Chemical fingerprints and structural keys are popular fingerprints for ML models and similarity searching, with RDKit topological fingerprinting (RDK) and ECFP4 classified as the former and Molecular Access Systems (MACCS) as the latter. This study calculated the RDK, ECFP4, and MACCS using the RDKit package in Python 3.7.3 (https://www.rdkit.org/). A total of 1024 RDK, 1024 ECFP4 and 166 MACCS fingerprint descriptors were calculated for each compound in the datasets, which were used as input for the ML models.

### ML model generation

**Fig. 2:**
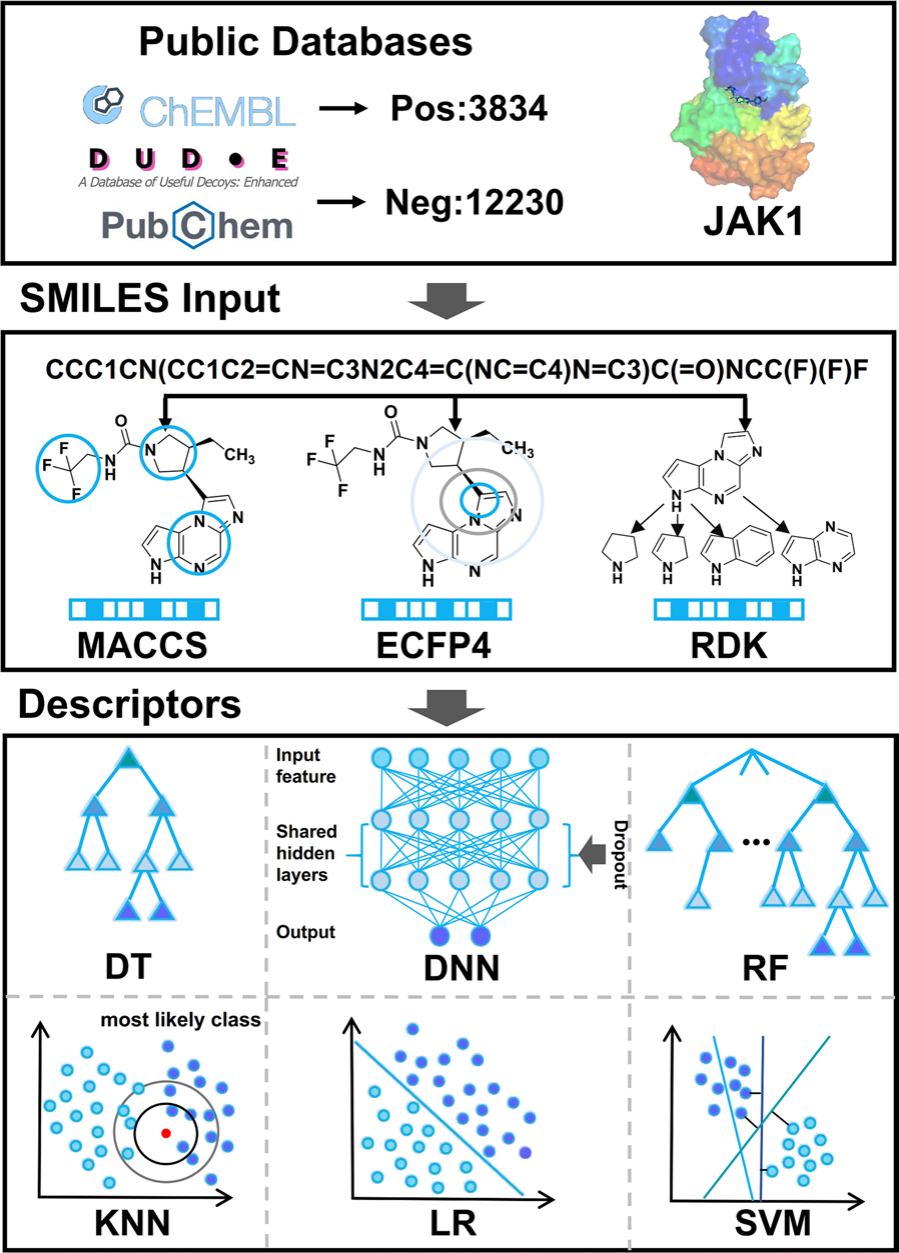
The flowchart for ML model construction

The Tensorflow 2.10.0 and Scikit-learn 1.0.2 packages in Python 3.7.3 were used to implement the DNN and the other ML models [34]. In this part, compounds in the datasets were binary classified by numerous algorithms with different fingerprint descriptors as input (listed below) (Fig. 2).

#### DNN model

Deep learning model with artificial neural networks as the architecture is a type of ML. As a framework of deep learning, the DNN was implemented to learn the molecular features in our study. The algorithm achieves data classification by performing a series of feature extractions and non-linear transformations on the input. Each neuron in the DNN receives outputs from the previous layer of neurons, multiplies them by weights, and propagates the result to the next layer of neurons, eventually generating the results. DNN of our method consisted of five fully-connected layers: one input layer, three hidden layers, and one output layer. Additionally, dropout was used to constrain each hidden layer to reduce overfitting.

#### SVM model

As a commonly used ML model, the SVM can be implemented by the SVM module of Scikit-learn. Through this algorithm, each data is mapped in an n-dimensional space, and then a hyperplane is estimated to optimally separate the compounds into active and inactive [35].

#### KNN model

The k-Nearest Neighbor (KNN) is an instance-based classification algorithm studying the class association of a data point in the feature space [36]. The KNN algorithm transforms input training data into a set of n-dimensional vectors in a multidimensional feature space. When a test vector is fed into the model, it can be assigned to the class that its k-nearest neighbors belong to at most, through the reference of Euclidean distances between the train and test vectors in the feature space. In this study, the Scikit-learn library was also used to implement the algorithm.

#### LR model

Logistic regression (LR) is another widely used supervised ML method. The algorithm allows predicting the probability of each compound to belong to the active or inactive group by mapping values between 0 and 1 as outputs through an activation function [37]. As a core part of the LR, a sigmoid function is used to realize the above process. The formula for LR is as follows:

$$ {\text{log}}\left[ {\frac{{\text{y}}}{{1 - {\text{y}}}}} \right] = {\text{b}}_{0} + {\text{b}}_{1} {\text{x}}_{1} + {\text{b}}_{2} {\text{x}}_{2} + {\text{b}}_{3} {\text{x}}_{3} + \cdots + {\text{b}}_{{\text{n}}} {\text{x}}_{{\text{n}}} $$

#### DT model

Decision Tree (DT) is a popular supervised ML method commonly used in both dataset classification and regression [38]. Therefore, the algorithm can also be applied to identify active compounds. With the tree’s structure to separate data, a leaf node, an internal node, a root node, and branches are included in the DT. As one of the standard algorithms used to solve classification problems, the Classification and Regression Tree (CART) algorithm can be applied to construct the DT model by slitting the nodes into sub-nodes on the basis of threshold values of attributes.

#### RF model

RF is an ensemble method that integrates diverse classifiers to make predictions for problems. Based on RF, ensemble learning can combine decision trees to yield better predictive performance than the other constituent classifiers. The RF can also prevent overfitting by selecting random subsets of training data for each tree and considering random factors at each decision node [39].

### ML model evaluation

#### Applicability domain

All models were developed on a limited number of compounds that do not cover the entire chemical space, and the applicability domain (AD) is the region of the chemical space where the models can accurately forecast new compounds. Principal component analysis (PCA) is a commonly utilized method for feature extraction through data dimensionality reduction. In this study, the optimal principal component (n) was determined by calculating the cumulative variance contribution. Subsequently, the value of n was employed for data dimensionality reduction, ultimately yielding the AD. The above process was implemented by the PCA function in Python’s Scikit-learn 1.0.2 package.

#### Evaluation metrics

To evaluate model quality, accuracy, precision, recall, F1 score, and Matthews correlation coefficient (Mcc) were calculated (Table 2) [40]. Moreover, AUC, an essential index for model quality assessment, was evaluated by the Python script.

**Table 2:** Description of the evaluation metrics

Evaluation metric	Equation
Accuracy	$\frac{TP + TN}{TP + FN + FP + TN}$
Precision	$\frac{TP}{TP + FP}$
Recall	$\frac{TP}{TP + FN}$
F1-score	$2*\frac{Precision*Recall}{Precision + Recall}$
Mcc	$\frac{TP*TN-FP*FN}{\sqrt{\left(TP + FP\right)\left(TP + FN\right)\left(TN + FP\right)\left(TN + FN\right)}}$

*TP* true positives, the number of correctly predicted active, *TN* true negatives, the number correctly predicted inactive, *FP* false positives, the number of incorrectly predicted active, *FN* false negatives, the number of incorrectly predicted inactive

#### Y-randomization

Y-randomization is a frequently used method to validate model robustness, which aims to test the random correlation between the dependent and independent variables. In this validation, the dependent variable Y is randomly ordered and a new model is built using the original independent variable matrix X. The process is repeated several times and each estimate of model accuracy and AUC are recorded. In total, 75% of the compounds in the training set were resampled and used for 500 runs of the Y-randomization test.

### VS of ZINC database

In this work, 1.6 million compounds were downloaded from the ZINC database (https://zinc.docking.org/). The RDKit package was used to calculate each compound’s molecular fingerprints. Finally, the ML model with optimal statistical parameters was selected for VS.

### Pharmacophore models generation and validation

#### Hiphop pharmacophore model

**Fig. 3:**
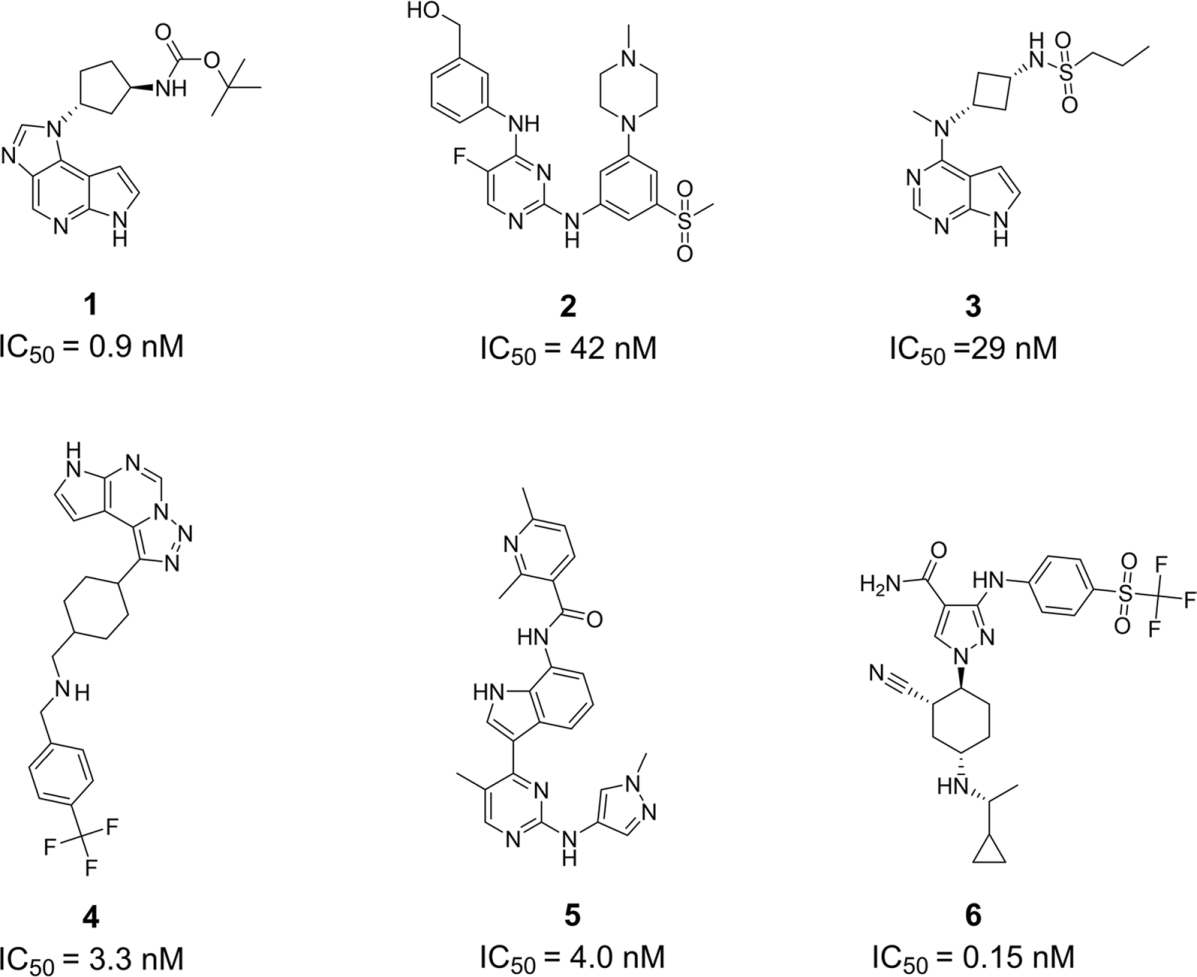
Chemical structures of the training set and their IC_50_ values (Hiphop)

The Hiphop algorithm mainly applies to constructing the critical common features from active ligands in the training set. Notably, the ‘Principle’ and ‘MaxOmitFeat’ values are important parameters to discriminate different inhibitors in the Hiphop model [41]. Both range from 0 to 2, the former with larger values correspond to a stronger activity of the inhibitor and the latter correspond to how many features can be missed. All the six JAK1 inhibitors included in the training set were obtained by literature search and energy minimized with Chemistry at Harvard Macromolecular Mechanics (CHARMm) force field [42–45] (Fig. 3). The ‘MaxOmitFeat’ value of 0 and the ‘Principle’ value of 2 were assigned to all molecules in the training set [46]. With six inhibitors as input, ten Hiphop pharmacophore models, which include hydrogen bond acceptor (A), hydrogen bond donor (D), aromatic feature (R), and hydrophobic feature (H), could be generated by the ‘common feature pharmacophore model generation’ protocol in Discovery Studio 2019.

#### Receptor-ligand pharmacophore model

Different with the Hiphop, the receptor-ligand pharmacophore models were constructed based on the description of receptor-ligand interactions [47]. By employing the ‘receptor-ligand pharmacophore generation’ module within Discovery Studio 2019, ten pharmacophore models, referred to as 6TPF 01–10, were derived from the crystal JAK1-ligand (PDB ID: 6TPF). In addition to the above pharmacophore feature, excluded volume spheres were also considered in the models to describe the interactions between ligands and receptors.

#### Pharmacophore model validation

To further assess the capability of models to discriminate inhibitors from database, a test database (including 3834 compounds from active dataset and 6590 compounds from DUD-E) was constructed to validate the pharmacophore models. We first examined the cutoff values by the SPSS 18.0 and then precision, recall, F1 Score, and Mcc were also calculated according to the formulas in Table 2.

The pharmacophore model with the optimal statistical parameters was implemented for further screening the hits obtained by the ML model.

### Molecular docking

X-ray crystal structure JAK1 receptor (PDB ID: 3EYG, resolution of 1.90 Å) was imported into Discovery Studio 2019 and prepared using the ‘Prepared Protein’ module, including removal of all the water molecules, insertion of missing loops, and addition of hydrogen atoms. The active site was defined as 12.0 Å around the endogenous ligand. The hits were energy minimized by the CHARMm force field [48]. The ligands and prepared protein were imported to implement CDOCKER docking with the parameters set as default. The interaction energy (CDOCKER interaction energy) with higher values corresponds to stronger binding between the ligands and proteins [49].

### MD simulation

MD simulations were performed in the GROMACS 2021.6. The software package and all simulation systems utilize the amber force field Amber99SB. Prior to that, the Multiwfn 3.8 (dev) and ORCA 5.0.2 software was used to calculate the resp charges of the ligands, and the Sobtop 1.0 (dev3.1) software was employed to convert it into a general Amber force field (GAFF) force field file that the GROMACS could recognize [50–52]. Then each complex was immersed in a water box with a 12 Å buffer of TIP3P water molecules and neutralized with an appropriate number of counterions (Na^+^ or Cl^–^). Firstly, energy minimization was performed with 1000 iterations of steepest descent and 5000 iterations of conjugate gradient algorithm. Thereafter, the NVT ensemble and NPT ensemble equilibration were carried out at 100 ps under 310 K [53]. In the simulation, the hydrogen bonds were constrained by the LINCS methods, the long-range electrostatic interactions were calculated using the PME method, and the short-range electrostatic and van der Waals interactions were truncated at a distance of 12.0 Å. MD simulations were performed for each of the systems for 50 ns at NPT conditions, and the integration time step was set to 2 fs and trajectories were recorded every 10 ps. Evaluation of molecular structure deviations and atomic flexibility during simulations were estimated using root mean square deviation (RMSD) and root mean square fluctuations (RMSF). Snapshots of these complexes were collected from the equilibrium region (30–50 ns) of the MD simulations. And the binding free energy was calculated by the Molecular Mechanics/Poisson-Boltzmann Surface Area (MM/PBSA) method. The specific formula is as follows:

$$ \begin{aligned} \Delta G_{bind} = & \,G_{complex} - \left( {G_{protein} + G_{ligand} } \right) \\ = & \,\Delta E_{MM} + \Delta G_{polar} + \Delta G_{nonpolar} - T\Delta S \\ = & \,\Delta H - T\Delta S \\ \end{aligned} $$

where G_complex_, G_protein_, and G_ligand_, represent the free energy of the complex, receptor, and ligand, respectively. ${\Delta E}_{MM}$ represents the gas phase interaction energy including van der Waals (${\Delta E}_{vdw}$) and electrostatic energy (${\Delta E}_{ele}$). ${\Delta G}_{polar}$ and ${\Delta G}_{nonpolar}$ represent the polar and nonpolar solvation free energy. $\Delta H$ corresponds to the enthalpy of bind, which is usually sufficient for comparing relative binding free energies of structurally similar ligands [54]. *− T∆S* is the entropy contribution, which was calculated using the interaction entropy (IE) method [55, 56].

### JAK1 kinase inhibition assay

The HTRF-based biochemical binding assay was performed to evaluate the inhibitory activities of the obtained compounds against JAK1. Briefly, different potential inhibitors and Tofacitinib were diluted and transferred to Gerinier white assay plate by echo, to get different concentration points in duplicate, which was followed by co-incubation for 30 min at room temperature. Then the Xl665 and antibody detection reagent mixture was added to each well, and the assay plate was incubated for 60 min at room temperature. After incubation, read TR-FRET signal 665/612 on Envision.

## Result and discussion

### Generation and evaluation of ML models

Eighteen ensemble models were obtained by applying different molecular fingerprints to the corresponding machine learning algorithms.

#### Applicability domain analysis

**Fig. 4:**
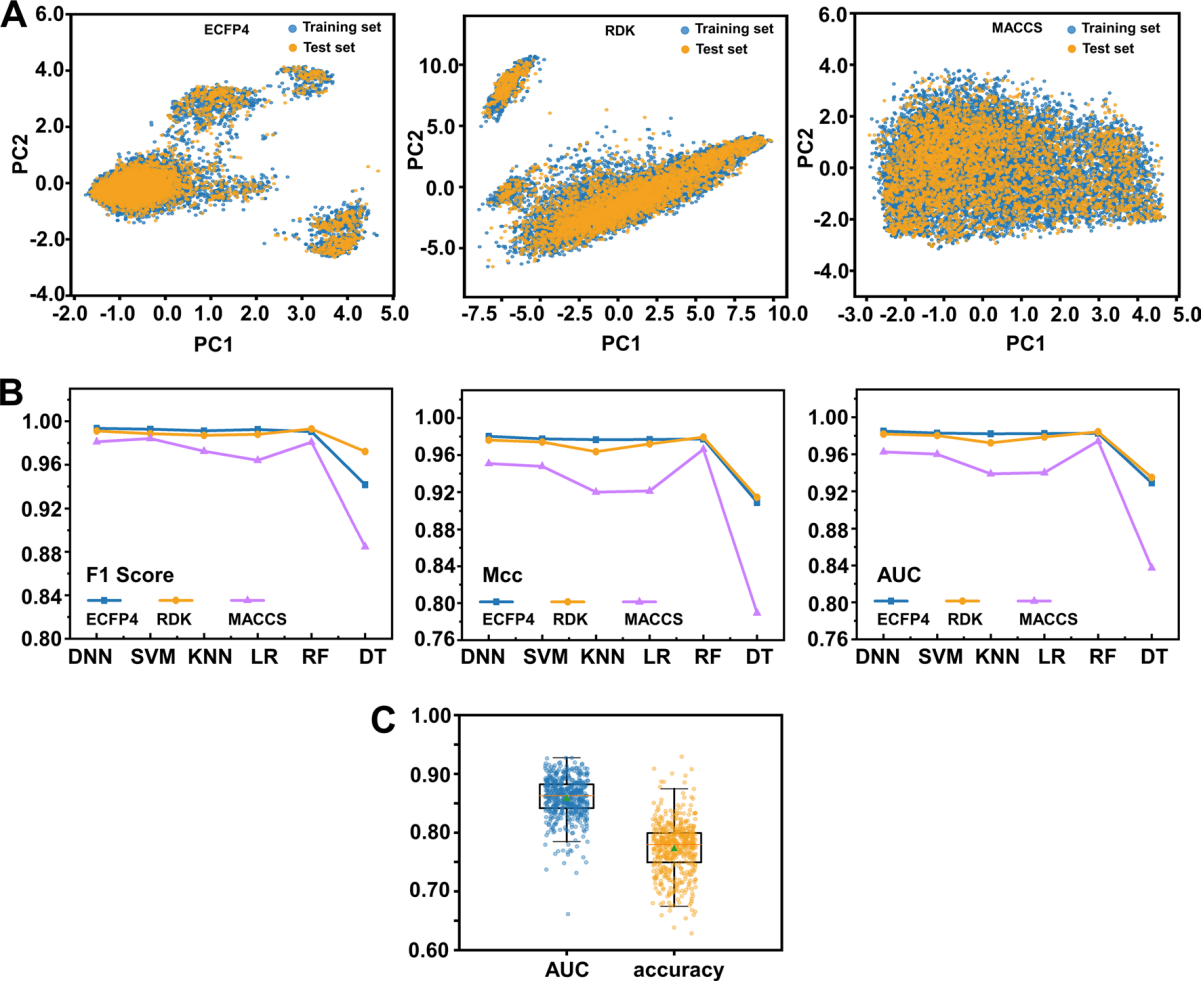
**A** Applicability domain plot based on ECFP4, RDK, and MACCS. **B** Comparison of the F1 Score, Mcc, and AUC of the different models. **C** The accuracy and AUC of Y-randomization models

Validation of Applicability domain (AD) was carried out on all 18 classification models mentioned above, with results depicted in Fig. 4A. The training set was denoted by blue, while the test set was represented by orange. It can be observed that these two sets exhibit a high degree of overlap in chemical space distribution. Furthermore, all the test set compounds were appropriately placed within the AD, signifying the performance of the prediction model was valid and worthy of trust.

#### Evaluation metrics analysis

The F1-score, Mcc, and AUC are the commonly used metrics for the overall discrimination ability to compare models [57]. Specifically, the statistical parameters of all the models were summarized in Table 3 and Fig. 4B. The following points can be noted: (1) All the 18 models showed the satisfactory performance to solve the binary classification problems, which were manifested in accuracy, precision, recall, F1-score, Mcc, and AUC values above 0.92, 0.86, 0.80, 0.83, 0.78, and 0.88, respectively. (2) In terms of fingerprint descriptors, ECFP4 performed slightly better than RDK, which clearly outperforms the MACCS. (3) Among six algorithms evaluated, DNN, RF, and SVM had stronger generalization ability than others, while the DT approach performed worst.

**Table 3:** Performance of the models based on different combinations of fingerprint descriptors and ML algorithms

	Training set		Test set					
	Accuracy	Precision	Accuracy	Precision	Recall	F1-score	Mcc	AUC
DNN								
ECFP4	0.9996	0.9983	0.9928	0.9764	0.9937	0.9850	0.9803	0.9935
RDK	0.9976	0.9976	0.9913	0.9743	0.9896	0.9819	0.9762	0.9911
MACCS	0.9978	0.9972	0.9818	0.9466	0.9791	0.9626	0.9508	0.9812
SVM								
ECFP4	0.9957	0.9956	0.9918	0.9763	0.9896	0.9829	0.9775	0.9928
RDK	0.9994	0.9976	0.9905	0.9762	0.9844	0.9803	0.9741	0.9886
MACCS	0.9905	0.9637	0.9803	0.9314	0.9906	0.9601	0.9478	0.9842
KNN								
ECFP4	0.9973	0.9948	0.9915	0.9874	0.9771	0.9822	0.9767	0.9913
RDK	0.9925	0.9744	0.9866	0.9575	0.9875	0.9723	0.9636	0.9871
MACCS	0.9855	0.9497	0.9696	0.9036	0.9771	0.9389	0.9200	0.9725
LR								
ECFP4	0.9992	0.9972	0.9915	0.9773	0.9875	0.9824	0.9768	0.9925
RDK	0.9995	0.9979	0.9898	0.9742	0.9833	0.9787	0.9720	0.9880
MACCS	0.9805	0.9567	0.9711	0.9305	0.9499	0.9401	0.9212	0.9641
RF								
ECFP4	0.9993	0.9976	0.9918	0.9894	0.9760	0.9827	0.9773	0.9905
RDK	0.9993	0.9972	0.9925	0.9884	0.9802	0.9843	0.9794	0.9930
MACCS	0.9995	0.9979	0.9878	0.9820	0.9666	0.9743	0.9663	0.9809
DT								
ECFP4	0.9745	0.9931	0.9674	0.9694	0.8916	0.9288	0.9091	0.9417
RDK	0.9696	0.9257	0.9686	0.9263	0.9437	0.9349	0.9143	0.9722
MACCS	0.9411	0.9127	0.9251	0.8697	0.8071	0.8372	0.7896	0.8848

When all of the statistical values of the different ML models were compared, the DNN-ECFP4, RF-RDK, and SVM-ECFP4 performed well in our binary classification problem. Collectively, the DNN-ECFP4 model exhibited the best predictive properties (the training precision was 0.9983; the precision, recall, F1-score, Mcc, and AUC of the test set were 0.9764, 0.9937, 0.9850, 0.9803, and 0.9935, respectively) and can be further used for large-scale VS of the ZINC database.

#### Y-randomization analysis

Finally, additional internal validation with the Y-randomization test was employed to test whether the best model DNN-ECFP4 was correlated by chance. The accuracy and AUC distribution results from 500 iterations were presented in Fig. 4C. Obviously, the correlation coefficient of the optimal model was significantly larger than that of the stochastic model, which indicates that there was a genuine link between the molecular characteristics defined by ECFP4 and compound activity, and that the optimal model was not a result of chance.

### DNN-based screening of the ZINC database

The ZINC database was used to identify potential inhibitors against the JAK1 receptor. For each compound, the ECFP4 fingerprint descriptor, as the input, was calculated and stored in CSV format using the RDKit package in Python. After creating a new data frame using the descriptor, potential hit compounds were screened using our DNN-ECFP4 model in the TensorFlow framework. The DNN-ECFP4 model could return the estimated probability (0 ≤ EstPGood ≤ 1) that a compound is in the active class, resulting in 13,976 molecules being identified to be potential JAK1 inhibitors (EstPGood > 0.5).

### Pharmacophore models generation and validation

#### Hiphop pharmacophore model

Six compounds against the JAK1 present in training set were employed to generate qualitative top 10 hypotheses using the Hiphop algorithm. Based on the pharmacophore feature similarities, three clusters were generated in this paper: Cluster I include two models with the combination of six pharmacophore chemical features like 1R, 2H, 1D, and 2A; Cluster II also has two models with the combination of six pharmacophore chemical features like 2H, 2D, and 2A; Cluster III includes six models with the combination of five pharmacophore chemical features like 1R, 1H, 1D, and 2A (Table 4).

**Table 4:** Performance of the pharmacophore models based on hiphop algorithms

No	Feature	Ranking score	Direct hit (DH)	Partial hit (PH)	Max fit	Cluster	Cutoff	Precision	Recall	F1 score	Mcc
Hiphop1	RHHDAA	91.652	111111	000000	6	I	2.3290	0.7839	0.06338	0.1173	0.1511
Hiphop2	RHHDAA	91.151	111111	000000	6	I					
**Hiphop3**	**HHDDAA**	**90.903**	**111111**	**000000**	**6**	**II**	**2.2359**	**0.9020**	**0.1080**	**0.1929**	**0.2377**
Hiphop4	HHDDAA	86.869	111111	000000	6	II					
Hiphop5	RHDAA	85.124	111111	000000	5	III	2.5917	0.6407	0.08790	0.1546	0.1305
Hiphop6	RHDAA	84.703	111111	000000	5	III					
Hiphop7	RHDAA	83.264	111111	000000	5	III					
Hiphop8	RHDAA	83.264	111111	000000	5	III					
Hiphop9	RHDAA	83.264	111111	000000	5	III					
Hiphop10	RHDAA	83.030	111111	000000	5	III					

The bold indicates the optimal model of different Hiphop models

**Fig. 5:**
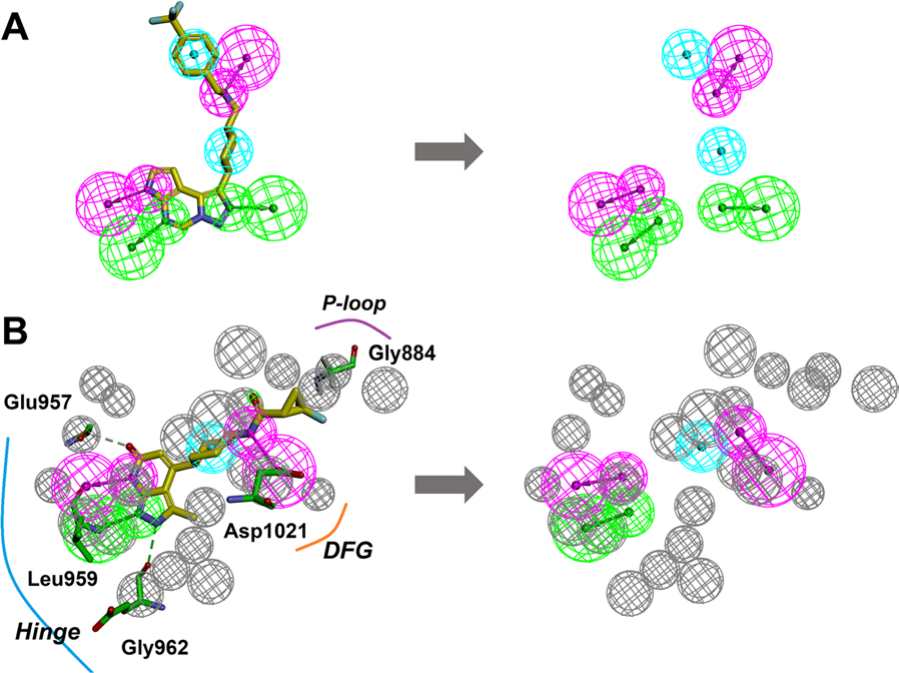
**A** The Hiphop3 pharmacophore model and it mapping with the compound **4**. **B** The 6TPF 08 pharmacophore model was identified based on the 6TPF complex. Green color indicates **A**; Cyan and magenta indicate **H** and **D**, respectively; Gray color indicates excluded volume

Cluster I-Hiphop1, Cluster II-Hiphop3, and Cluster III-Hiphop5, which was the highest rank score for each cluster, were employed as 3D queries to identify active compounds in the test database. As a result, each pharmacophore model captured partial molecules and assigned the ‘Fitvalue’ to each compound. To further analyze the quality of the pharmacophore model from a statistical perspective, cut-off values, as well as metrics such as precision, recall, F1 Score, and Mcc, were calculated and presented in Table 4. Based on the validation results, Hiphop3 (precision = 0.9020, F1 Score = 0.1929, and Mcc = 0.2377) was able to discriminate active molecules from inactive molecules more effectively using 2.2359 as cutoff values. The Hiphop3 model, including 2H, 2D, and 2A pharmacophore features, shows the best alignment with the compound **4** which has shown in Fig. 5A.

#### Receptor-ligand pharmacophore model

From the Protein Data Bank (https://www.rcsb.org/), the co-crystal structure (PDB ID: 6TPF) has been derived and checked to abstract and interpret the mutual interactions between receptor and ligand. As critical starting point, the active pocket of 6TPF was visualized and further analyzed. The ligand could be embedded well in the active site, while the pyrazolopyridinone docked the vicinity of the hinge region and formed hydrogen bonds with Glu 957 and Leu 959. In addition, the NH and difluorocyclopropyl group in the ligand docked near DFG and P-loop in the receptor. The ‘Receptor-Ligand pharmacophore generation’ protocol was used to derive ten pharmacophore models called 6TPF 01–10. The models generated contains D points towards Leu 959, Glu 957 and Asp 1021, A points towards Leu 959, some hydrophobic feature and excluded volume spheres, which recapitulates the mutual interactions between receptor and ligand well.

Similarly, the external test set was employed to identify the pharmacophore models, and the multiple statistical parameters of each model are manifested in Table 5. The 6TPF 08 model with a probability cutoff of 1.5159 has been selected for screening as it exhibits optimal statistical parameters, including precision of 0.8152, F1 score of 0.3547, and Mcc of 0.3131. In addition, the model consists of two D points towards leu 959 and Asp 1021, one A point towards Leu 959, one hydrophobic feature, and 23 excluded volume spheres (Fig. 5B).

**Table 5:** Performance of the different receptor-ligand pharmacophore models

No	Feature	Selectivity score	Cutoff	Precision	Recall	F1 score	Mcc
6TPF 01	DDDHH	10.828	0.000507	0.7736	0.03208	0.06161	0.1047
6TPF 02	ADDHH	9.9146	0.6751	0.8260	0.07799	0.1425	0.1802
6TPF 03	DDDH	9.3133	1.0060	0.7778	0.02556	0.04949	0.09404
6TPF 04	DDDH	9.3133	1.0049	0.7904	0.08163	0.1480	0.1742
6TPF 05	DDHH	8.3998	1.3878	0.7175	0.1067	0.1857	0.1744
6TPF 06	DDHH	8.3998	2.0498	0.8541	0.09468	0.1705	0.2079
6TPF 07	DDHH	8.3998	2.4278	0.6014	0.06573	0.1185	0.09915
**6TPF 08**	**ADDH**	**8.3998**	**1.5159**	**0.8152**	**0.2267**	**0.3547**	**0.3131**
6TPF 09	ADDH	8.3998	1.55616	0.6935	0.1328	0.2229	0.1859
6TPF 10	ADHH	7.4863	1.4717	0.6248	0.2572	0.3644	0.2251

The bold indicates the optimal model of different receptor-ligand pharmacophore models

### Pharmacophore models-based screening

With the 13,976 hits as input, the ‘Build 3D database’ protocol generated different conformations of each hit in Discovery Studio 2019. Then the Hiphop3 and 6TPF 08 pharmacophore models were employed as 3D queries to screen above molecules further with the search method of best. According to the results, the screening process using the Hiphop3 model only yielded 254 molecules (Fitvalue > 2.2359). Additionally, 972 molecules matched all the chemical features of 6TPF 08 (Fitvalue > 1.5159). Overall, 113 compounds could simultaneously match the pharmacophore features of the two models. Further analysis revealed that all 113 compounds are also located within the AD of the DNN-ECFP4 model (Additional file 1: Fig. S1).

### Docking and visual inspection

Molecular docking allows a visual understanding of protein–ligand interactions at the molecular level to evaluate the stability and binding affinity of their docking complexes. Prior to docking, the endogenous ligand Tofacitinib was extracted from JAK1 (PDB ID:3EYG; resolution 1.90 Å) crystals and redocked into the active pocket of this receptor. The RMSD value of 1.25 Å was calculated for the re-docked pose with respect to the co-crystallized ligand, which was below the 2.00 Å threshold, confirming the accuracy of the docking protocols and parameters. The 113 compounds obtained from the previous step were docked into the active pocket of the JAK1 protein [58]. To screen out molecules that fit into the protein active pocket well, the following criteria have been applied: (i) The shape between the protein’s active pocket and ligand is complementary; (ii) The main skeleton of the hit compound could dock at the vicinity of the hinge region and formed the hydrogen bonds with Glu957 and Leu959; (iii) Calculated CDOCKER interaction energy of hit compound was less than − 35 kcal mol^−1^. A total of 13 compounds (i.e., 11.5%) fulfilled the above criteria (Table 6). In general, the CDOCKER interaction energy of the hit compounds ranged from − 38.98 – − 71.20 kcal mol^−1^, mostly higher than that of the co-crystal ligand Tofacitinib (− 45.19 kcal mol^−1^), suggesting that they have a high binding affinity for JAK1.

**Table 6:** Chemical structures and CDOCKER interaction energy of the screened hits and Tofacitinib

Compound	ZINC ID	Structure	CDOCKER interaction energy kcal mol^−1^	RO5
Z1	ZINC000585263163		− 71.2019	0
Z2	ZINC000257214054		− 60.2629	0
Z3	ZINC000257238169		− 60.2221	0
Z4	ZINC000253536640		− 59.3322	0
Z5	ZINC000952973512		− 51.7868	0
Z6	ZINC000071639668		− 49.4108	0
Z7	ZINC000067713616		− 48.0285	0
Z8	ZINC000299785860		− 47.0708	0
Z9	ZINC000019766606		− 46.772	0
Z10	ZINC001506420991		− 45.3281	0
Z11	ZINC000005740776		− 43.6171	0
Z12	ZINC000952972597		− 42.7699	0
Z13	ZINC000072410164		− 38.9812	0
Tofacitinib	Tofacitinib		− 45.1924	0

RO5: Besides the Lipinski’s rule of five

**Fig. 6:**
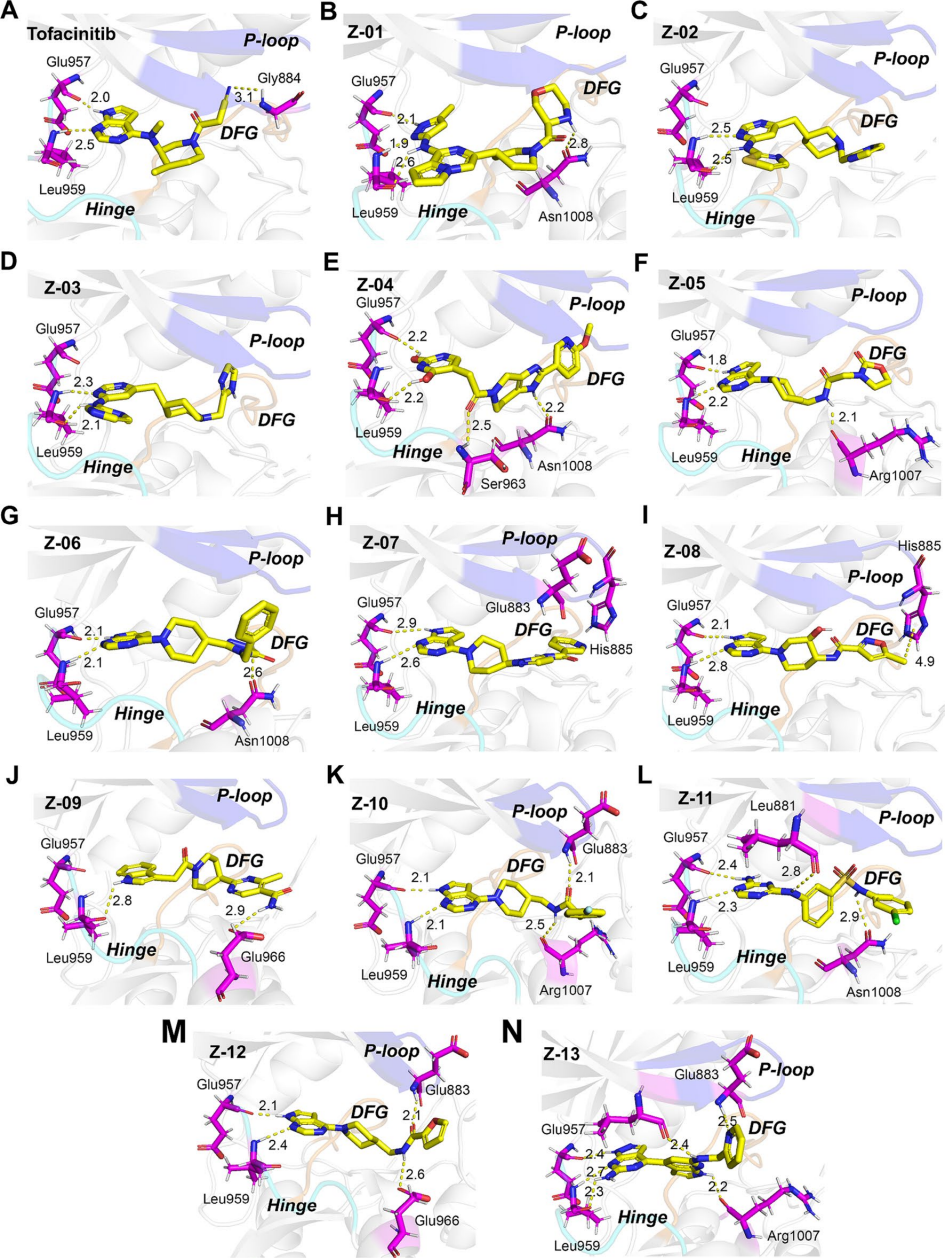
**A**–**N** Binding mode of the Tofacitinib and top 13 compounds in the active site of JAK1 (PDB ID: 3EYG)

Moreover, all the 13 hit compounds and Tofacitinib could dock into the JAK1 active site and mainly form the hydrogen bonds with Leu881, Glu957, Leu959, Ser963, Glu966, Arg1007 and Asn1008. The roles of most residues have been reported in previous studies [42, 59, 60]. Tofacitinib assumed a favorable conformation within the active pocket of JAK1, with its t-butyl group directed towards the P-loop and its pyrimidopyrrole moiety buried in the vicinity of hinge region (Fig. 6A). Z-01, exhibiting the lowest CDOCKER interaction energy, was sandwiched within the active site and oriented such that its main skeleton was buried in a deep hydrophobic pocket and its morpholine group pointed towards the P-loop (Fig. 6B). Additionally, the Z-01 could form four hydrogen bonds with Glu957, Leu959 of the hinge and Asn1008. The ligands Z-02 and Z-03 demonstrated binding modes similar to that of Z-01 (Fig. 6C-D). Compound Z-08 shared analogous binding modes with the Tofacinitb, Z-04, Z-05 and Z-07. Its pyrimidopyrrole core docked near the hinge region and formed stable hydrogen bonding with Glu957 and Leu959 at a bond distance of 2.1 Å and 2.8 Å, respectively (Fig. 6I). Furthermore, its methyl group pointed towards the P-loop and formed Pi-alkyl interactions with His885 at a bond distance of 4.9 Å. With the fluorobenzene ring oriented towards the solvent-exposed region, the ligand Z-10 could be inserted into the ATP-binding cleft of JAK1 and establish hydrogen bonding with Glu957, Leu959, Arg1007, and Glu883 at a bond distance of 2.1 Å, 2.1 Å, 2.5 Å, and 2.1 Å, respectively (Fig. 6K). Moreover, Z-06, Z-09, Z-11, Z-12, and Z-13 displayed the comparable binding modes to that of Z-10 (Fig. 6G, J, L-N). Finally, the swissADME (http://www.swissadme.ch/) was employed to calculate the ADME values of the screened compounds, and all 13 compounds meet Lipinsk’s rule (RO5 = 0) (Table 6, Additional file 1: Table S1).

### MD simulations analysis

MD simulations and molecular docking have been considered two complementary strategies for understanding the mutual interactions between receptors and ligands [61]. MD simulations can verify the plausibility of docking results and monitor the time-resolved motion of macromolecules. We carried out MD simulations of the 13 ligand–protein docking complexes mentioned above and Tofacitinib-protein based on the same simulation parameters to evaluate the stability of the complex interactions.

**Fig. 7:**
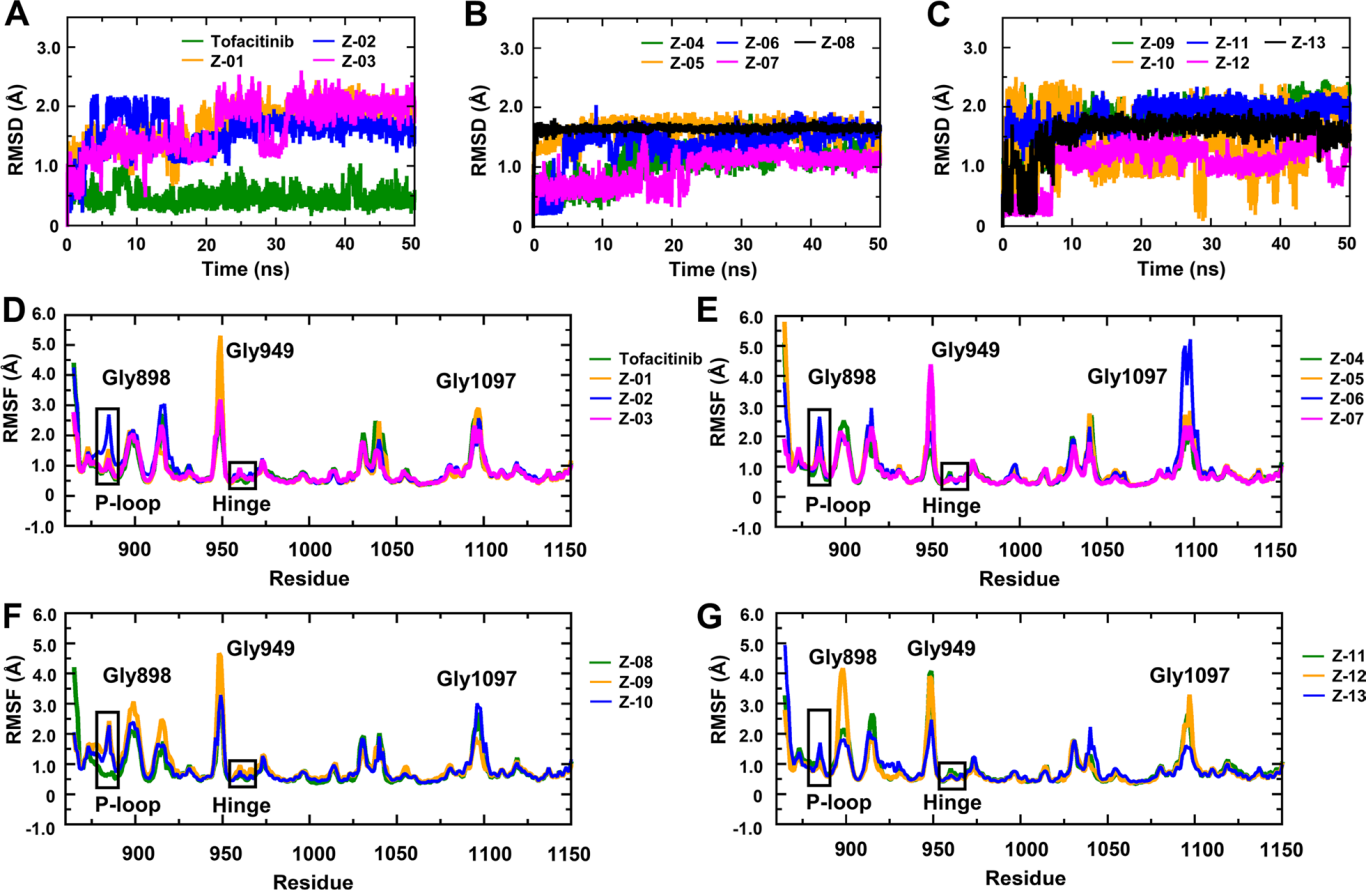
**A**–**C** RMSD plots of the Tofacitinib and top 13 compounds bound to the JAK1 protein. **D**–**G** RMSF plots of the proteins in the 14 systems

The RMSD between initiated docked poses and ligands in the 14 systems was calculated and plotted against time (Fig. 7A–C). In the initial phase, marginal variations in most systems had been noticed. However, the fluctuations in the ligand trajectories smoothed out after 10 ns (20 ns for Z-01 and Z-03), and all complexes had RMSD values of less than 2.5 Å (Tofacitinib < 1.0 Å; Z-02, 04–08,12–13 < 2.0 Å), indicating that they were stably binding in the hydrophobic pocket of JAK1. Of these, compound Z-08 had the minimum fluctuation after converging, whereas Z-10 showed the maximum fluctuation. Further, trajectory analysis of the ligands was conducted to explore ligand conformation changes during MD simulation. After undergoing a certain degree of relative conformational change, the hydroxyl group on the furan ring of Z-08 could form a stable hydrogen bond with Asp1021 in the DFG, generating a stable pose of Z-08 in the active site with low fluctuation (Additional file 1: Fig. S2A). The binding core of Z-10 exhibited high stability in the hinge region, while the terminal phenyl ring displayed significant flexibility with conformational changes over time. Furthermore, Z-10 alternately approached Gly883 and Arg1007 during the process of conformational changes. Despite the considerable fluctuation in the ligand’s RMSD values, the Z-10 remained relatively stable in the protein’s active site during the simulation (Additional file 1: Fig. S2B).

The RMSF of the Cα atoms in 14 MD trajectories was computed to characterize the flexibility of the protein during the simulation. Generally, the flexibility of amino acid residues, especially those near the active pocket, could decrease after the formation of the ligand–protein complex, which in turn reflects the binding affinity between protein and ligand. The RMSF plots of the 14 complexes are given in Fig. 7D–G. Overall, the RMSF plots of 13 hits followed a similar trend to that of Tofacitinib. Besides, the residues around the active site (e.g., 956–964 and 1019–1024), especially the binding residues, demonstrated higher stability, whereas P-loop and other loops (895–903, 946–951, and 1094–1099) in which Gly898, Gly949 and Gly1097 are located experienced a dramatic tensile deformation. Specifically, the critical residues from the hinge region displayed low flexibility, with RMSF values of less than 1.0 Å. Compared to the other complexes, contraction at the P-loop made the region more stable in the Z-08-protein, Z-12-protein, and Tofacitinib-protein which could be explained by the result of the molecular docking study. Additionally, the Z-12-protein complex increased residues Gly898’s flexibility and experienced a dramatic stretching deformation in this region with the RMSF > 4.0 Å, while the other complexes’ RMSF < 3.0 Å. In contrast, the binding of Z-04 and Z-06 to the JAK1 protein induced the enhancement of molecular rigidity at the loop in which Gly949 is located. For the Z-06-protein complexes, the loop in which Gly1097 is located exhibited high flexibility with the RMSF values of more than 4.5 Å.

**Fig. 8:**
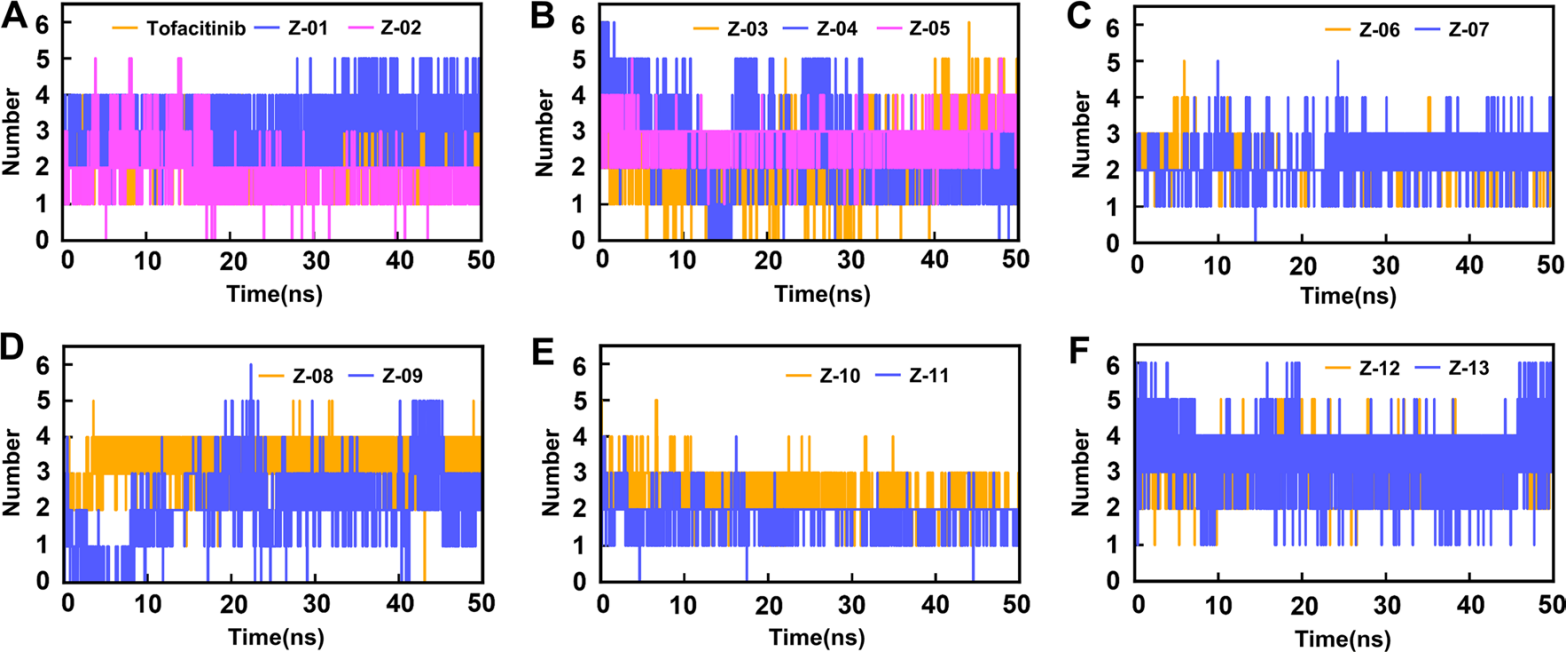
**A**–**F** H-bond plots of the Tofacitinib and top 13 compounds in the MD simulations

As hydrogen bonding interaction (H-bond) is an essential contributor to ligand–protein binding, the number of H-bond was also evaluated during the dynamical shift of the compound at the active site (Fig. 8). The average number of H-bond forming between Z-13 and JAK1 protein was more than that of other compounds with the value of four. Besides, on average, Z-01, Z-04, Z-05, Z-08, Z-10, and Z-12 formed three hydrogen bonds during the dynamical shift, while that of the other compounds was two.

The above results fully confirmed that there were constantly stable hydrogen bonds and no major conformational changes in the ligands and key residues during the MD simulation, suggesting the reliability and stability of the docking study.

### Binding free energy analysis

To analyze the binding affinities of 13 hits, the MM-PBSA method was employed to calculate the binding free energies (ΔG_bind_) of the systems. As shown in Table 7, the free binding energy of Tofacitinib was − 89.375 kJ mol^-1^, which was inferior to Z-1, Z-2, Z-13 and superior to the other compounds. Besides, the ΔG_bind_ of compounds Z-01, Z-02, Z-08, Z-10, Z-13, and Tofacitinib in JAK1 were less than − 80 kJ mol^−1^, while that of the other compounds were less than − 40 kJ mol^−1^. The results implied that firmly binding complexes could be formed between each compound and JAK1, while Z-01, Z-02, Z-08, Z-10, and Z-13 might have a stronger binding ability than the other compounds. In all 14 systems, ΔE_vdw_, ΔE_ele_, and ΔG_nonpolar_ (negative values) contributes to binding affinities between ligand and protein, whereas the ΔG_polar_ provides an unfavourable contribution to the total binding free energy. Interestingly, the ΔG_nonpolar_ of all the 14 systems were numerically similar. The polar solvation and electrostatic interactions can compensate for each other in a vacuum. Considering the sum of the ΔE_ele_ and ΔG_polar_, the value of Z-07 was significantly higher than that of the other compounds, which is unfavorable for the total binding free energy. Furthermore, in absolute value the ΔE_vdw_ of Z-03 and Z-09 was relatively low with respect to the other compounds, which therefore contributes less to ΔG_bind_. Hence, the van der Waals interaction, polar solvation interaction and electrostatic interaction might significantly contribute to the binding affinities of these compounds.

**Table 7:** Complex combined with free energy analysis (kJ mol^−1^)

Compound	Contribution						
	ΔE_vdw_	ΔE_ele_	ΔG_polar_	ΔG_nonpolar_	ΔH	− TΔS	ΔG_bind_
Z1	− 199.489	− 61.975	166.983	− 28.080	− 122.561	25.872	− 96.689
Z2	− 193.448	− 34.742	132.523	− 24.187	− 119.854	18.897	− 100.957
Z3	− 169.821	− 41.000	156.956	− 24.674	− 78.539	19.144	− 59.395
Z4	− 183.974	− 99.026	235.543	− 26.234	− 73.692	26.635	− 47.057
Z5	− 172.621	− 97.908	201.274	− 24.643	− 93.898	27.021	− 66.877
Z6	− 182.998	− 61.215	177.037	− 25.070	− 92.246	19.823	− 72.423
Z7	− 205.951	− 95.615	251.211	− 28.001	− 78.356	17.335	− 61.021
Z8	− 187.935	− 99.731	206.722	− 23.972	− 104.917	23.975	− 80.942
Z9	− 154.846	− 83.911	202.570	− 27.465	− 63.652	22.671	− 40.981
Z10	− 163.668	− 63.231	144.840	− 24.021	− 106.081	23.762	− 82.319
Z11	− 195.285	− 74.995	208.557	− 25.102	− 86.825	21.468	− 65.357
Z12	− 188.001	− 68.133	186.627	− 23.413	− 92.920	20.049	− 72.871
Z13	− 187.556	− 42.403	140.100	− 24.713	− 114.573	18.434	− 96.139
Tofacitinib	− 193.829	− 101.702	203.954	− 24.559	− 116.136	26.761	− 89.375

Besides, the per-residue energy decomposition of the top 9 hits with higher binding free energy was presented in Additional file 1: Fig. S3. and the Val889, Glu957, Phe958, Leu959, and Leu1010 can be identified as critical residues by combining these data with the docking results.

### Evaluation of JAK1 inhibitors’ kinase activity

**Fig. 9:**
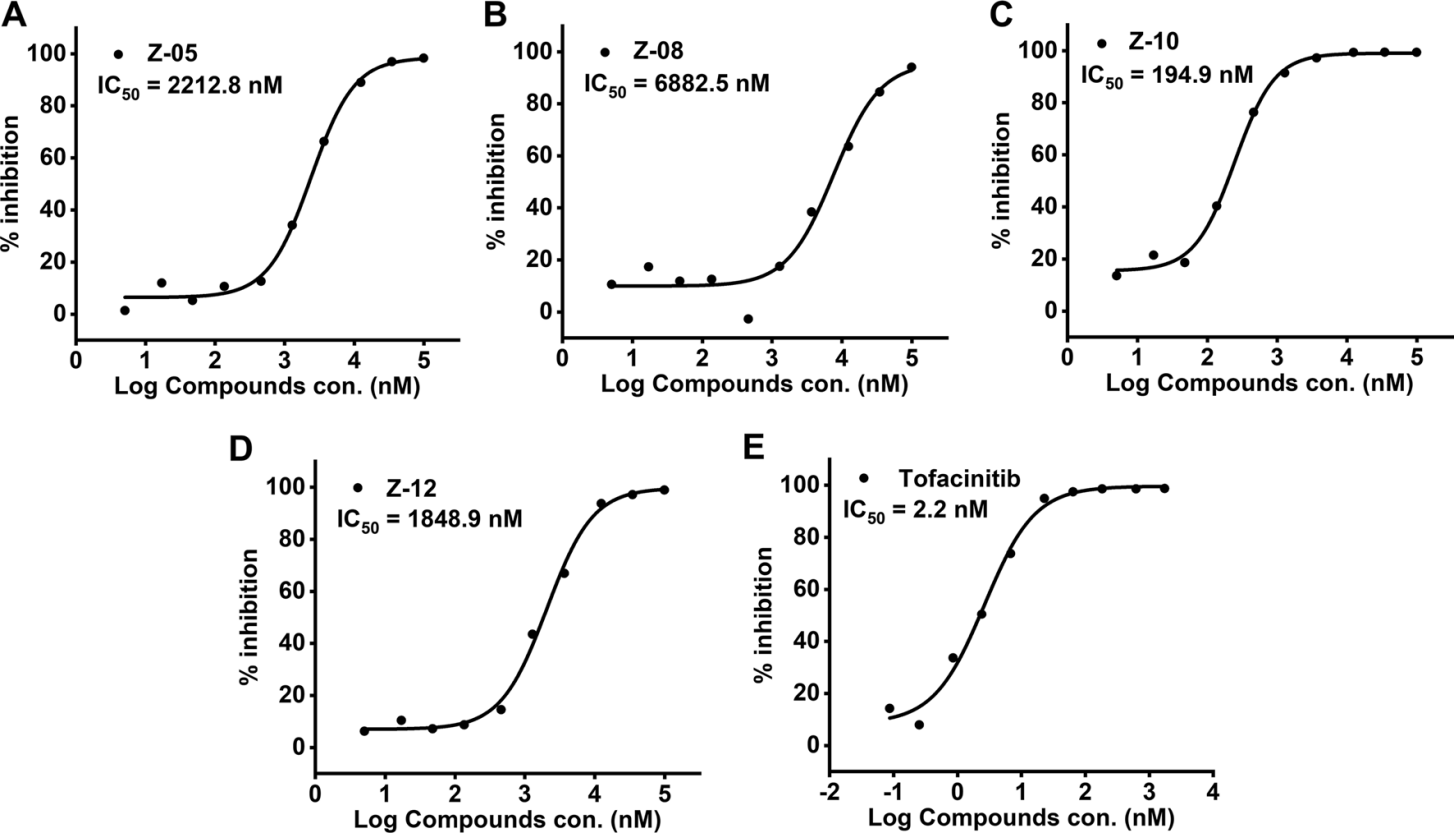
**A**–**E** IC_50_ of Z-05, Z-08, Z-10, Z-12, and Tofacitinib toward the JAK1

To further verify the screening results, four purchasable compounds (Z-05, Z-08, Z-10, Z-12) were obtained from ChemBridge for the kinase assay (Additional file 1: Table S2). As presented in Fig. 9, the JAK1 kinase inhibition assay revealed the Z-10 as the best inhibition activity against JAK1 (IC_50_ = 194 nM) among all the tested compounds. The kinase assay results further strengthened these compounds’ ability to inhibit the JAK1 kinase activity and the reliability of our screening method.

## Conclusion

In our study, a dataset consisting of 3834 JAK1 inhibitors and 12,230 decoys was collected, and 18 classification models were constructed using a combination of three molecular descriptors and six ML algorithms. When comparing between the different descriptors and algorithms, the classification effect of ECFP4 and RDK was close and noticeably stronger than that of MACCS; DNN, RF, and SVM had the stronger generalization ability than the other algorithms. The best classifier DNN-ECFP4 based on DNN and ECFP4 achieved an accuracy of 0.9928 and an AUC of 0.9935 for the test set. Furthermore, two pharmacophore models were constructed and identified based on different algorithms. Combining the DNN-ECFP4 model and the pharmacophore models, the ZINC database was screened, followed by further selection based on CDOCKER to hit the top 13 compounds. The MD and free energy calculation were employed to further confirm the interaction strength and stability of all 13 hits and the receptor. Moreover, we demonstrated the enzyme inhibition activities of purchasable compounds in vitro. As a result, all purchasable compounds Z-05, Z-08, Z-10, and Z-12 exhibited more potent inhibitory activity against JAK1 (IC_50_ < 10,000 nM). Significantly, IC_50_ of the most active compound Z-10 against JAK1 was 194.9 nM. In this study, the DNN model exhibited notably higher screening efficiency compared to traditional pharmacophore and molecular docking screening methods. Incorporating the DNN model as the initial step of the screening program and combining it with subsequent screening steps can significantly enhance both the rate and scope of screening. Besides, the hit compounds can be further studied, including the in vitro/vivo studies and structural modification.

### Supplementary Information

**Additional file 1: Table S1.** The ADMET parameters of the screened hits. **Table S2.** The information of four purchasable compounds. **Figure S1. **The AD of DNN-ECFP4 model and the chemical space of hits. **Figure S2.** The initial (pink sticks) and final (yellow sticks) conformations of compounds Z-08 (**A**) and Z-10 (**B**). **Figure S3.** The graph of the binding free energy decomposition per residue for JAK1-inhibitor complexes.

## Data Availability

The authors confirm that the data supporting the findings of this study are available within the article [and/or] its additional file.
