# Supplementary material for: Discovery of novel JAK1 inhibitors through combining machine learning, structure-based pharmacophore modeling and bio-evaluation
Source: J Transl Med. 2023 Aug 28;21:579. doi: 10.1186/s12967-023-04443-6 (PMC10464202; doi:10.1186/s12967-023-04443-6)
Supplement: Supplementary file 1 — Additional file 1: Table S1. The ADMET parameters of the screened hits. Table S2. The information of four purchasable compounds. Figure S1. The AD of DNN-ECFP4 model and the chemical space of hits. Figure S2. The initial (pink sticks) and final (yellow sticks) conformations of compounds Z-08 (A) and Z-10 (B). Figure S3. The graph of the binding free energy decomposition per residue for JAK1-inhibitor complexes. [file 12967_2023_4443_MOESM1_ESM.docx]

***Supplementary materials***

**Discovery of novel JAK1 inhibitors through combining machine learning, structure-based pharmacophore modeling and bio-evaluation**

Table S1. The ADMET parameters of the screened hits.

|  | Mw | Donors | acceptors | rotatable bonds | Log Po/w |
| --- | --- | --- | --- | --- | --- |
| Z1 | 409.48 | 3 | 5 | 5 | 2.63 |
| Z2 | 355.46 | 2 | 5 | 6 | 2.19 |
| Z3 | 350.42 | 2 | 6 | 6 | 1.58 |
| Z4 | 382.37 | 3 | 8 | 5 | 1.29 |
| Z5 | 358.39 | 2 | 5 | 6 | 2.19 |
| Z6 | 375.43 | 2 | 4 | 4 | 2.42 |
| Z7 | 388.43 | 2 | 5 | 5 | 2.13 |
| Z8 | 342.35 | 3 | 6 | 4 | 1.55 |
| Z9 | 377.44 | 2 | 4 | 5 | 2.20 |
| Z10 | 367.42 | 2 | 4 | 6 | 2.66 |
| Z11 | 376.82 | 3 | 5 | 5 | 1.96 |
| Z12 | 325.37 | 2 | 4 | 5 | 2.27 |
| Z13 | 357.37 | 4 | 5 | 4 | 1.20 |
| Tofacinitib | 311.38 | 1 | 3 | 4 | 1.87 |

Table S2. The information of four purchasable compounds.

| Compound | Supplier | | | |
| --- | --- | --- | --- | --- |
|  | ID | Structure | SaltForm | StereoChemistry |
| Z-05 | ChemBridge  26679346 |  | free | racemic |
| Z-08 | ChemBridge  47610722 |  | free | trans |
| Z-10 | ChemBridge  54048714 |  | HCOOH | achiral |
| Z-12 | ChemBridge  46379544 |  | free | achiral |

**
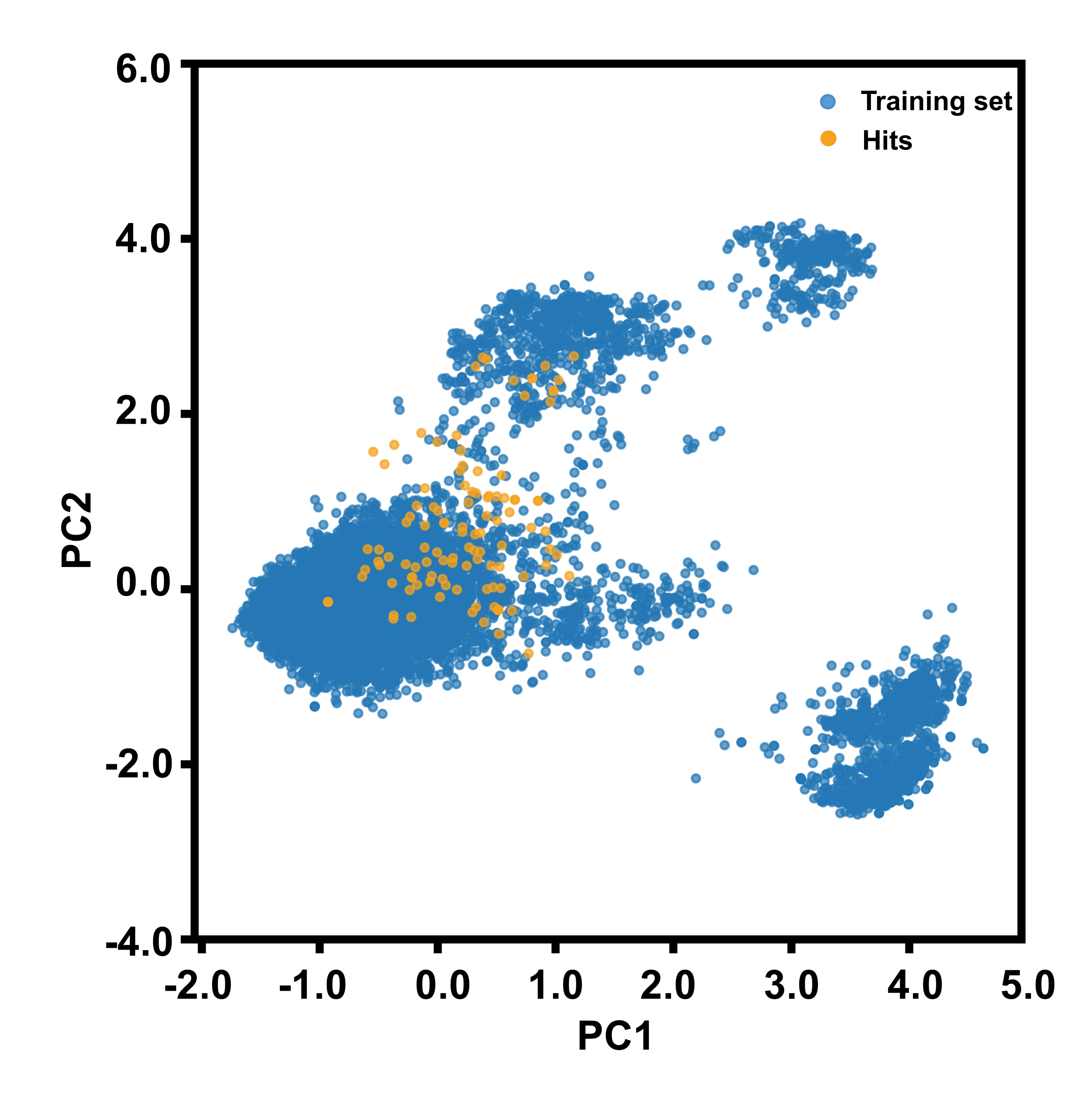
**

Fig. S1. The AD of DNN-ECFP4 model and the chemical space of hits

**
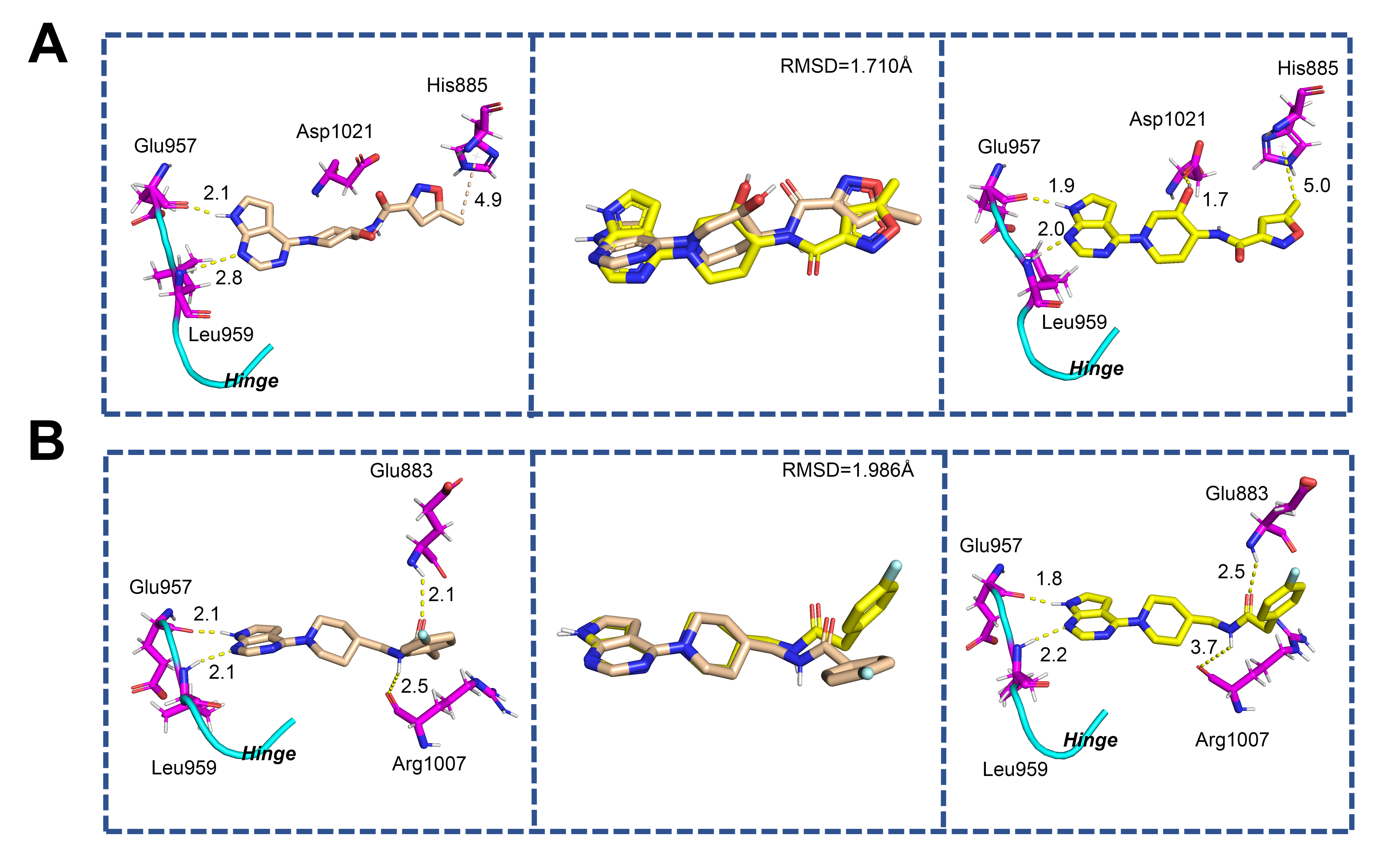
**

Fig. S2. The initial (pink sticks) and final (yellow sticks) conformations of compounds Z-08 (A) and Z-10 (B)


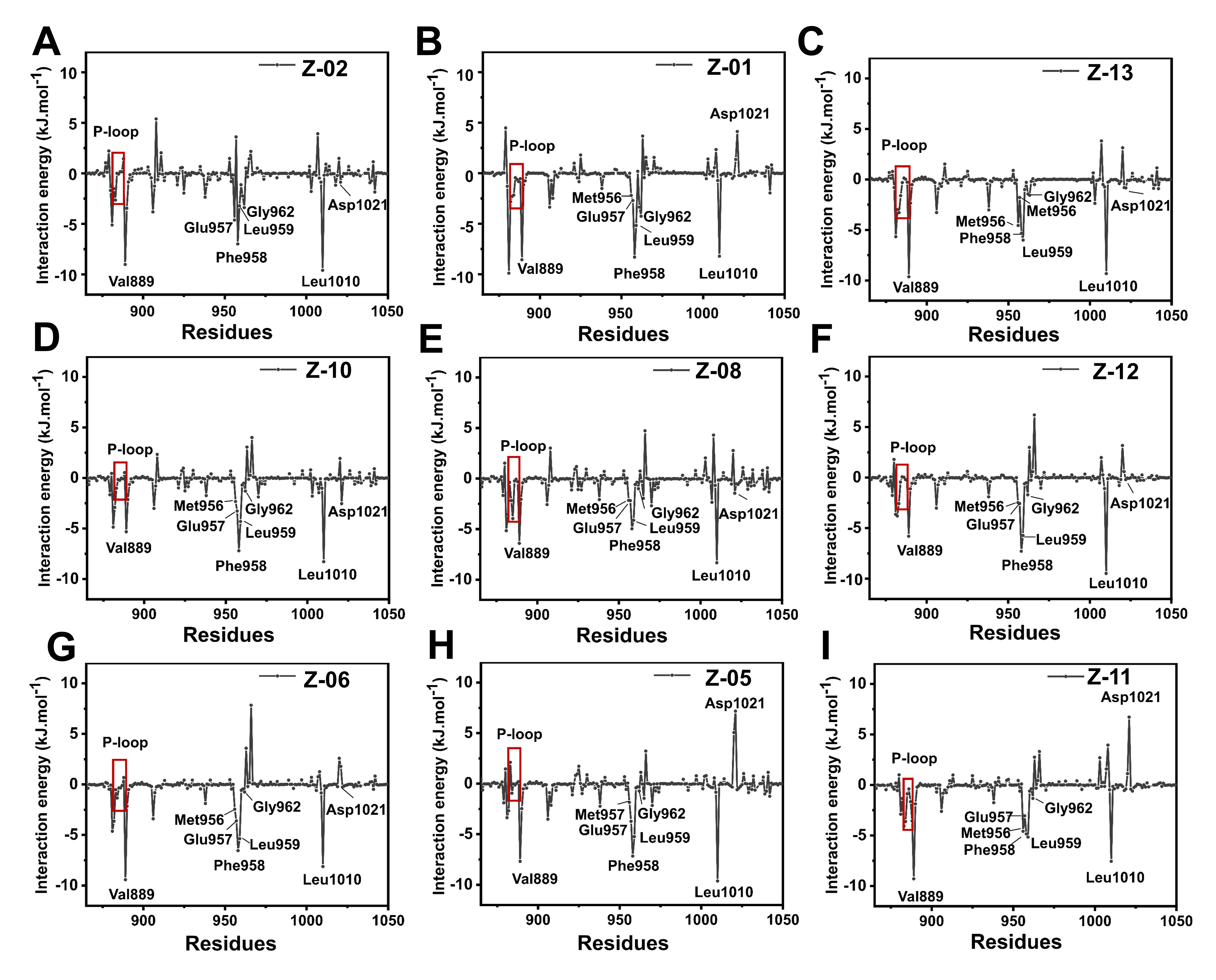


Fig. S3. The graph of the binding free energy decomposition per residue for JAK1-inhibitor complexes.

**Note:** The machine learning algorithm utilized the following hardware and software components: a 4 cores processor, 16 GB of RAM, the Tensorflow 2.10.0 and Scikit-learn 1.0.2 packages for Python 3.7.3 Additionally, Discovery Studio 2019 was employed for pharmacophore modeling and molecular docking. It is worth noting that GROMACS 2021.6, utilized for molecular dynamics simulations, required higher computing power. Specifically, running 50 ns on the 96 cores utilized in this study took approximately 12 hours.
